# Establishment and application of a multiple cross displacement amplification combined with nanoparticles-based biosensor method for the detection of *Bordetella pertussis*

**DOI:** 10.1186/s12866-020-01945-x

**Published:** 2020-08-24

**Authors:** Shijun Li, Chunting Liu, Ying Liu, Qing Ma, Yue Wang, Yi Wang

**Affiliations:** 1 Laboratory of Bacterial Infectious Disease of Experimental Center, Guizhou Provincial Center for Disease Control and Prevention, Guiyang, 550004 Guizhou China; 2 Department of Respiratory Disease, Beijing Pediatric Research Institute, Beijing Children’s Hospital, Capital Medical University, National Center for Children’s Health, Beijing, 10045 PR China; 3 Key Laboratory of Major Diseases in Children, Ministry of Education, Beijing Key Laboratory of Pediatric Respiratory Infection Disease, National Clinical Research Center for Respiratory Diseases, Beijing Children’s Hospital, Capital Medical University, National Center for Children’s Health, Beijing, 10045 PR China

**Keywords:** *Bordetella pertussis*, Multiple cross displacement amplification, Lateral flow biosensor, MCDA- LFB, Detection limit

## Abstract

**Background:**

*Bordetella pertussis* is the causative agent of pertussis, a respiratory tract infectious disease*.*

Efficient techniques for detection of *B. pertussis* isolates are important for clinical diagnosis.

Multiple cross displacement amplification (MCDA), a novel isothermal amplification based molecular detection method, has been developed to overcome the technical drawback of the current methods in recent years. This aim of this study is to develop a MCDA with Nanoparticles-based Lateral Flow Biosensor (MCDA-LFB) for the detection of *B. pertussis.*

**Results:**

A set of 10 primers based on the pertussis toxin (PT) promoter region sequence of *B. pertussis* was designed. The *B. pertussis*-MCDA-LFB assay was successfully established and optimized at 64 °C for reaction of 40 min. The detection limit was determined as 10 fg/reaction of pure DNA, and no cross-reactions to non-*B. pertussis* strains were observed, based on the specificity validation. The whole operation, ranging from template preparation to result reporting, could be completed within 70 min without requirement of costly equipment. The *B. pertussis*-MCDA-LFB in clinic sample detection yielded identical positive rates with traditional culture and showed higher sensitivity than conventional PCR. The results of MCDA-LFB are easier to read due to the usage of LFB.

**Conclusions:**

The isothermal amplification based MCDA-LFB established in the present study is a specific, sensitive, rapid and economical technique for the detection of *B. pertussis*.

## Background

Pertussis, also referred whooping cough or named as “cough of 100 days”, is a respiratory tract infectious disease primarily leaded by the causative agent *Bordetella pertussis* [1], a gram negative bacterium which can attach to the ciliated cells and colonize the human upper respiratory tract [2]. It can damage the respiratory epithelium by producing multiple toxins and leads to systemic effects such as the lymphocytosis [3], local inflammatory changes in the mucosal lining of the respiratory tract [1]. Whooping cough can be life threatening for newborns and young children without vaccination. *B. pertussis* can also infect other population such as adults, and a shift of infection cases from school-age children to adolescents, adults and infants has been observed in the last decade [2, 3]. Although the efficacious pertussis vaccines has been widely used, whooping cough results in about 200,000 deaths annually and at least 24 million new pertussis cases were reported in 2014 in children under 5 years old [2]. Most deaths caused by pertussis are from developing countries, with majority of cases in children under the age of 5 months [4]. According to the estimation of WHO in 2013, *B. pertussis* infection caused about 60,257 deaths in children younger than 5 years [5].

The appropriate use of clinically accurate diagnostic tests is essential for the effective diagnosis of pertussis. Pertussis can be diagnosed by the commonly used culture and serological methods [6]. However, 7 to 10 days are needed to isolate and identify, *B. pertussis* isolates using conventional culture method, while the indirect serological diagnosis needs about 1 month based on the reaction with sera, acute- and convalescent-phase sera [7]. As a result of the shortage of culture and serologic diagnosis, the detection results are usually not available even till the patient has already recovered from the illness. In order to improve the detection duration and sensitiveness of laboratory diagnosis, PCR-based methods have been used for the detection of *B. pertussis*. Most available diagnostic PCR assays, including nested PCR and real-time PCR, merely developed based on fewer targets, which cannot differentiate *B*. *pertussis* from other *Bordetella* spp. including *B*. *holmesii* [8]. For example, real time-PCR based on part of insertion sequences (IS) cannot be used for specific species detection, because IS481 also exists in the genome of some *B. bronchiseptica strains and B. holmesii* strains while IS1001 is proved in some *B. bronchiseptica* and *B. parapertussis* (Martini, et al., 2017). Other targets including sequence of BP*283*, BP*485*, *ptxS1* and the pertactin genes have been used for the detection of *B. pertusis*, but cross-reactivity with other *Bordetella* spp. have been reported [9]. It has been reported that the pertussis toxin (PT) promoter sequences differ from the sequence of *B. pertussis*, *B. parapertussis* and *B. bronchiseptica* [10, 11], which enable the feasibility to develop PCR based methods targeting the PT promoter region for the specific detection of *B. pertusiss*. Previous study reported that PCR based on the PT promoter sequence specifically recognize *B. pertussis*, but the sensitivity is insufficient [12, 13]. More recently, loop-mediated isothermal amplification (LAMP) targeting the PT promoter region has been established to amplify the DNA of *B. pertussis* with high degree of simplicity and specificity [7]. Although LAMP assays showed high efficiency of amplification samples with marginal amounts of DNA were still difficult to detect [14]. Particularly, the results of LAMP techniques for *B. pertusiss* detection were determined using agarose gel electrophoresis, real-time turbidity equipment or color indicator. The operation of gel electrophoresis for the analysis of products is complicated, and the risk of DNA product degradation and carryover contamination is increased [15]. The reading of real-time turbidity of *B. pertussis*-LAMP amplification requires optical instrument, and is easily to be interfered by the background. The judgment of result of *B. pertussi*-LAMP using the naked eyes is possibly subjective, which is also possible that the result reading be ambiguous to the naked eye due to the low concentration of DNA template.

In recent years, multiple cross displacement amplification (MCDA), a novel isothermal amplification based molecular detection method, has been developed to overcome the technical drawback of the current methods [16, 17]. MCDA designed ten primers (two displacement primers, two core primers and six amplification primers), instead of two in PCR and six in loop-mediated isothermal amplification assay, to recognize 10 different regions of target sequence, which enhance its sensitivity, specificity, and shorten its reaction time. Moreover, MCDA assay requires merely isothermal conditions and simple equipment such as water bath or heater. The amplification products can be detected by using disposable lateral flow biosensors, with visual result judgment [18], which is objective and does not need any instrument for result reading. MCDA has been validated for the effective detection of various bacteria such as *Klebsiella pneumonia*, *Staphylococcus aureus and Pseudomonas aeruginosa* [16, 17]. However, MCDA-LFB has not been validated for the detection of *B. pertussis*. Herein, MCDA targeting the PT promoter region, combined with nanoparticles-based biosensor, was established and optimized for simple, rapid, high specific and sensitive detection of *B. pertussis* at the current report, which was further evaluated by comparison with traditional culture and PCR methods.

## Results

### Confirming the effectiveness of *B. pertussis*-MCDA primer set

**Fig. 1:**
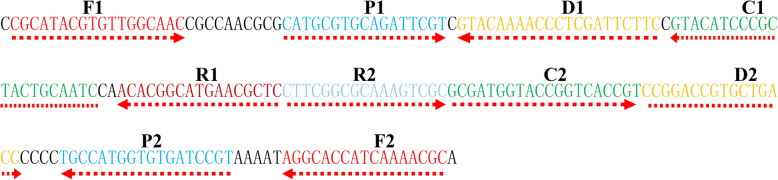
Primer sequences and the location of the PT promoter region *of B. pertussis* used for the MCDA assay in this study. The diagram shows the target sequence and primer design for the MCDA assay. The nucleotide sequence of the sense strand of the PT promoter region of *B. pertussis* was displayed. Right arrows indicate sense sequence. Left arrows indicate complementary sequence

**Fig. 2:**
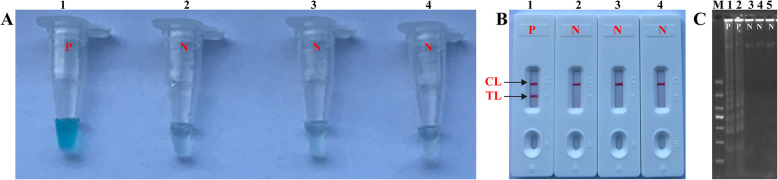
Effectiveness of *B. pertussis*-MCDA primer set. **a** Products of the *B. pertussis*-MCDA amplification were visually monitored by determination of the color change using the malachite green. **b** visual detection of *B. pertussis-*MCDA products using lateral flow biosensor. Tube 1/Biosensor 1: positive results of *B. pertussis* strain ATCC-9340; Tube 2/Biosensor 2: negative results of *S. aureus*; Tube 3/Biosensor 3: negative results of *N. meningitidis*; Tube 4/Biosensor 4: DW (blank control). TL: test line; CL: control line. **c** The target-specific formation of MCDA products was confirmed by electrophoresis gels images. M: DNA Marker; 1 and 2: positive results of *B. pertussis* strain ATCC-9340; 3: negative results of *S. aureus*; 4: negative results of *N. meningitides*; 5: DW (blank control)

To confirm the effectiveness of *B. pertussis*-MCDA primer set targeting on the PT promoter region (Fig. 1, Table 1), the DNA extracted from *B. pertussis* strain was amplified with MCDA at 64 °C for 1 h. Both the colorimetric indicator (MG) and LFB showed that DNA of *B. pertussis* strain ATCC-9340 was amplified effectively, but no amplification of DNA products was observed for *S. aureus* (GZCDC isolate), *N. meningitidis* (GZCDC isolate) and DW (blank control) (Fig. 2a and b). The electrophoresis gels images, showing a DNA ladder, were observed in positive reaction, but no DNA ladder was observed in the negative and blank controls (Fig. 2c). Therefore, the MCDA primer set was selected as the candidate to establish MCDA-LFB assay for the detection of *B. pertussis*.

**Table 1:** The primers used in this study

Primers name ^**a**^	Sequences and modifications ^**b**^	Length ^**c**^	Gene
F1	5′- CGCATACGTGTTGGCAAC-3’	18 nt	PT promoter region
F2	5′-GCGTTTTGATGGTGCCT-3’	17 nt	
CP1	5′-GATTGCAGTAGCGGGATGTACCATGCGTGCAGATTCGT −3’	38 mer	
CP2	5′-GCGATGGTACCGGTCACCGTACGGATCACACCATGGC-3’	37 mer	
C1	5′-GATTGCAGTAGCGGGATGTAC-3’	21 nt	
C1*	5′-Biotin-GATTGCAGTAGCGGGATGTAC-3’	21 nt	
C2	5′-GCGATGGTACCGGTCACCGT-3’	20 nt	
D1	5′-GAAGAATCGAGGGTTTTGTAC-3’	21 nt	
D2	5′-CCGGACCGTGCTGACC-3’	16 nt	
R1	5′-GAGCGTTCATGCCGTGT-3’	17 nt	
R1*	5′-FITC-GAGCGTTCATGCCGTGT-3’	17 nt	
R2	5′-CTTCGGCGCAAAGTCGC-3’	17 nt	

^a^ C1*, 5′-labeled with biotin when used in MCDA-LFB assay; R1*, 5′-labeled with FITC when used in MCDA-LFB assay. ^b^ *FITC* fluorescein isothiocyanate, ^c^ *mer* monomeric unit, *nt* nucleotide, *PT* pertussis toxin

### Optimizing the reaction temperature of *B. pertussis*-MCDA-LFB

**Fig. 3:**
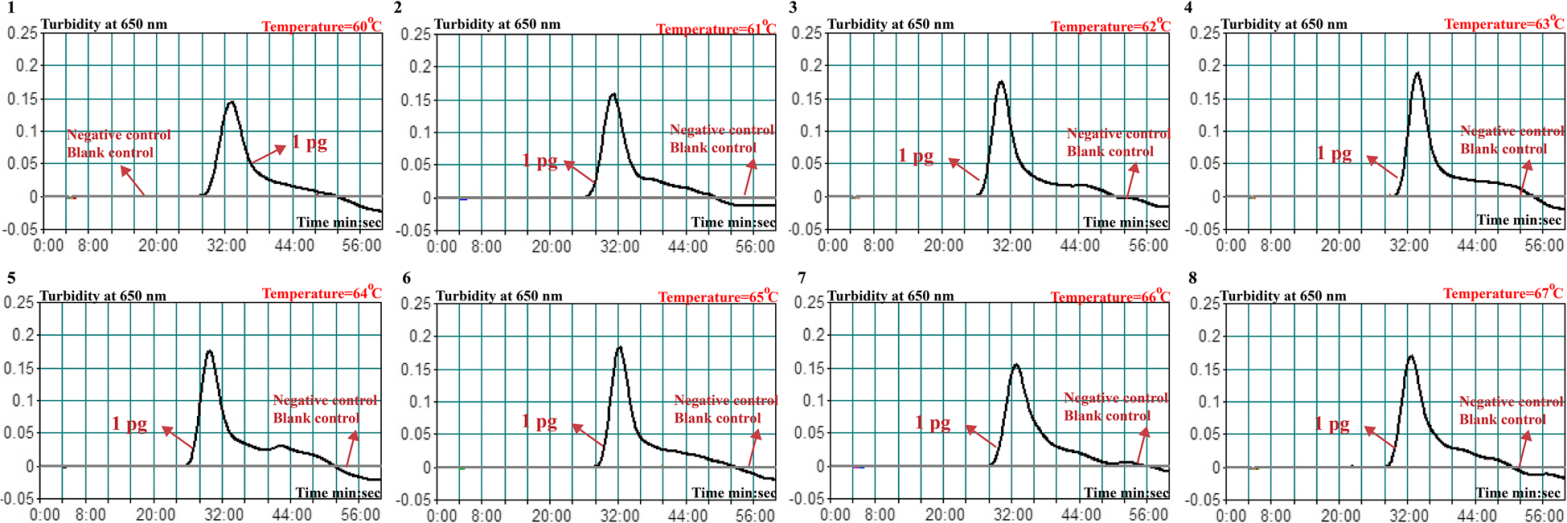
Optimization of the reaction temperature for *B. pertussis*-MCDA-LFB assay. The real-time turbidity of MCDA amplification for the detection of *B. pertussis* was monitored, and the concentrations of DNA were shown with corresponding curves as indicated in the figures. The threshold value was 0.1 and a turbidity > 0.1 was defined as positive. Eight of kinetic graphs (1–8) were generated at different temperatures points (from 60 to 67 °C with interval of 1 °C.) with DNA template with 10 pg each reaction. Graphs 2–8 showed strong amplification

To optimize amplification temperature, genomic DNA templates from *B. pertussis* ATCC-9340 were applied as the positive control with 10 pg DNA for each reaction. The real-time turbidity of amplification products was monitored. Typical kinetics graphs were observed for all the determined temperatures (from 60 to 67 °C with increments of 1 °C), and faster amplification was achieved at 64 °C (Fig. 3). Therefore, 64 °C was selected as the amplification temperature for the further experiments.

### Sensitivity of MCDA-LFB for *B. pertussis* detection

**Fig. 4:**
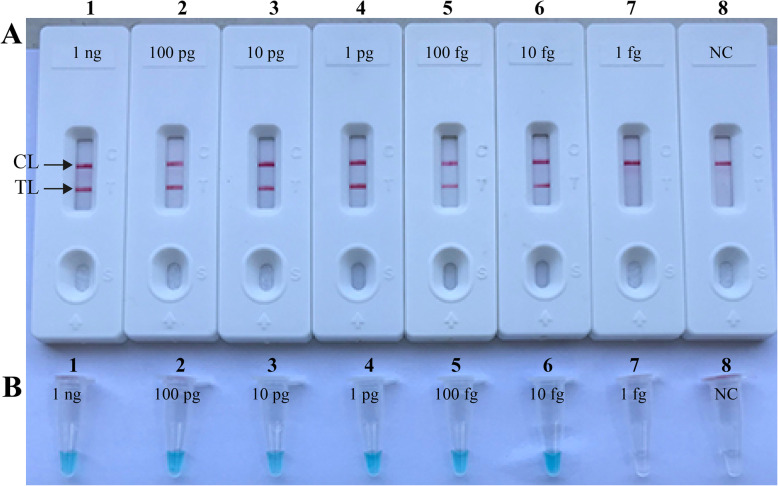
Sensitivity evaluation of *B. pertussis*-MCDA-LFB assay for the detection of DNA from *B. pertussis* strain ATCC-9340. Lateral flow biosensor (**a**) and colorimetric indicator (**b**) were used to monitor the reaction products. Biosensors (**a**)/Tubes (**b**) from 1 to 7 indicate 1 ng, 100 pg, 10 pg, 1 pg, 100 fg, 10 fg, and 1 fg of target DNA; Biosensor 8 indicate DW (negative control), respectively. Amplification of 1 ng, 100 pg, 10 pg, 1 pg, 100 fg and 10 fg of DNA template showed positive amplification, while the 1 fg per reaction and blank control was negative. NC: negative control; TL: test line; CL: control line

Serially diluted genomic DNA from *B. pertussis* was used to examine the detection limit of the *B. pertussis*-MCDA-LFB. The results detected with LFB showed that the sensitivity of the *B. pertussis*-MCDA-LFB was as low as 10 fg (2.4 copies) per reaction (Fig. 4a). By MG, the detection limit of the *B. pertussis*-MCDA assay was also 10 fg per reaction (Fig. 4b), which was in accordance with the detection results of LFB.

### Optimal reaction time of *B. pertussis*-MCDA-LFB

**Fig. 5:**
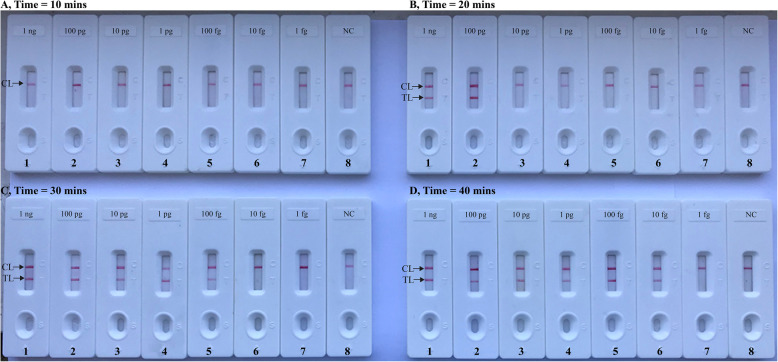
The optimal reaction time for *B. pertussis*-MCDA-LFB detection. Four different reaction times including 10 min (**a**), 20 min (**b**), 30 min (**c**), and 40 min (**d**) were examined and compared at 64 °C. Biosensors 1–7 indicate 1 ng, 100 pg, 10 pg, 1 pg, 100 fg, 10 fg, 1 fg of DNA extracted from *B. pertussis,* respectively; Biosensor 8 indicates DW (blank control),. The optimal sensitivity appeared at 40 min of amplification (**c**). TL indicates test line; CL indicates control line

In order to optimize the reaction time of *B. pertussis*-MCDA-LFB assay, different time span (10, 20, 30 and 40 min) were chosen for amplification at 64 °C based on the standard conditions of MCDA. The lowest DNA level (10 fg of *B. pertussis* DNA templates per reaction) showed both TL and CL lines when the reaction only continued for 40 min at temperature of 64 °C. Therefore, 40 min was selected as the optimal amplification time for *B. pertussis*-MCDA-LFB (Fig. 5).

### Specificity of *B. pertussis*-MCDA-LFB assay

**Fig. 6:**
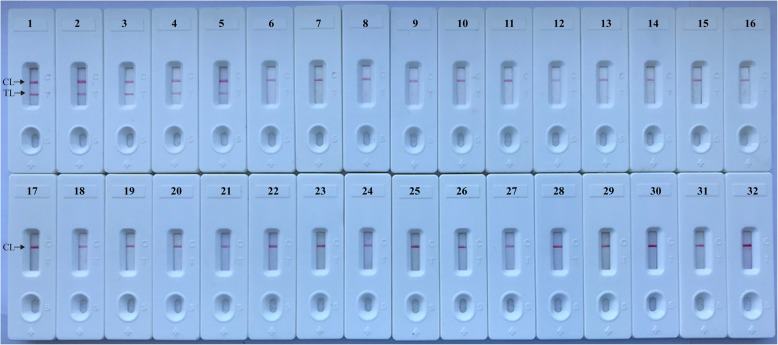
Specificity assessment of the *B. pertussis*-MCDA-LFB Assay. Different genomic DNA templates were applied for *B. pertussis*-MCDA-LFB specificity evaluation. Biosensor 1 used DNA template from *B. pertussis* strain ATCC-9340; Biosensors 2–5 used DNA templates from *B. pertussis* strain (GZCDC isolate), respectively. Biosensor 6 used DNA template from *Bordetella parapertussis.* Biosensors 7–31 used DNA template from bacterial strain of *Neisseria meningiditis, Hemophililus parainfluenza*, *Streptococcus pneumoniae, Klebsiella pneumoniae, Pseudomonas aeruginosa, Mycoplasma pneumoniae, Legionellae bacillus, Acinetbacter baumannii, Staphylococcus aureus, Staphylococcus saprophyticus, Salmonella,* enteropathogenic *E. coli*, enterotoxigenic *E. coli*, invasive *E.coli*, enterohemorrhagic *E. coli*, enteroaggregative *E.*coli, *Streptococcus suis*, *Vibrio cholerae*, *Enterococcus faecalis, Enterococcus faecium, Bacillus* proteus, E*nterobacter cloacae; Listeria monocytogenes*, *Shigella flexneri*, *Shigella boydii,* respectively*.* Biosensor *32* used DW as negative control

The initial specificity of the MCDA primers for *B. pertussis* detection was confirmed by blast analysis in NCBI data base and the PT promoter sequence were only found in *B. pertussis* strains, with similarity of 99.5% ~ 100%. Then, genomic DNA template from *B. pertussis* and non-*B. pertussis* strains (Table 2) were used to assess the specificity of *B. pertussis*-MCDA-LFB Assay. The results showed that two red lines appeared at the location of TL and CL on the strips for the *B. pertussis* strain, but only one line appeared at the location of CL for all the non-*B. pertussis* strains and blank control (Fig. 6), suggesting negative results for non-*B. pertussis* bacterial isolates and DW sample (blank control).

**Table 2:** Bacterial strains used in this study

No.	Bacteria	Serogroup/ Species	Strain name. (source of strain) ^**a**^	No. of strains
1	*Bordetella pertussis*	U	ATCC9340	1
2	*B*ordetella *pertussis*	U	Isolated strain (GZDC)	16
3	*Bordetella parapertussis*	U	Isolated strains (GZDC)	2
4	*Neisseria meningiditis*	U	Isolated strains (GZDC)	1
5	*Hemophililus parainfluenza*	U	Isolated strains (GZDC)	1
6	*Streptococcus pneumoniae*	U	Isolated strains (GZDC)	1
7	*Klebsiella pneumoniae*	U	Isolated strains (GZDC)	1
8	*Pseudomonas aeruginosa*	U	Isolated strains (GZDC)	1
9	*Mycoplasma pneumoniae*	U	Isolated strains (GZDC)	1
10	*Legionellae bacillus*	U	Isolated strains (GZDC)	1
11	*Acinetbacter baumannii*	U	Isolated strains (GZDC)	1
12	*Staphylococcus aureus*	U	Isolated strains (GZDC)	1
13	*Staphylococcus saprophyticus*	U	Isolated strains (GZDC)	1
14	*Salmonella*	Typhimurium	Isolated strains (GZDC)	1
15	enteropathogenic *E. coli*	U	Isolated strains (GZDC)	1
16	enterotoxigenic *E. coli*	U	Isolated strains (GZDC)	1
17	invasive *E.coli*	U	Isolated strains (GZDC)	1
21	enterohemorrhagic *E. coli*	U	EDL933	1
18	enteroaggregative *E.*coli	U	Isolated strains (GZDC)	1
19	*Streptococcus suis*	U	Isolated strains (GZDC)	1
20	*Vibrio cholerae*	U	Isolated strains (GZDC)	1
21	*Enterococcus faecalis,*	U	ATCC35667	1
22	*Enterococcus faecium*	U	Isolated strains (GZDC)	1
23	Bacillus proteus	U	Isolated strains (GZDC)	1
24	*Enterobacter cloacae*	U	Isolated strains (GZDC)	1
25	*Listeria monocytogenes*,	4a	ATCC19114	1
26	*Shigella flexneri*,	F1a	Isolated strains (GZDC)	1
27	*Shigella boydii*	U	Isolated strains (GZDC)	1

^a^ *U* unidentified serotype, *ATCC* American Type Culture Collection

### Application of *B. pertussis*-MCDA-LFB in clinical samples

In order to evaluate the *B. pertussis*-MCDA-LFB for clinical application, 67 nasal swab samples were used for DNA template preparation, which were were applied for the evaluation of *B. pertussis*-MCDA-LFB in clinical application. The detection results demonstrated 31 samples were *B. pertussis* positive when using RT-PCR for detection, while the other 36 samples were proved negative. MCDA-LFB detection demonstrated 37 positive samples, which included the 31 positive samples detected with real-time PCR. Besides, MCDA-LFB showed consistent results with traditional cultivation and isolation methods in the clinic sample detection, both of which confirmed 37 positive clinic samples (Table 3).

**Table 3:** Comparison of real-time PCR, culture-biotechnical and MCDA-LFB for the detection of *Bordetella pertussis* in clinical samples

Detection methods	samples (*n* = 67)	
	Positive	Negative
Real-time PCR ^a^	31	36
Culture	37	30
MCDA-LFB	37	30

^a^ The real-time PCR was designed using the PT promoter region of *B. pertussis*

## Discussion

It has been reported that 156 million cases of pneumonia each year in children younger than 5 years are estimated by the World Health Organization (WHO) [19, 20]. Recent evidence shows a sizable infant cases of pertussis present with acute pneumonia and 2% of clinical pneumonia cases of enrolled infants were caused by pertussis [20, 21]. *B. pertussis* is the major causative reagent of pertussis, albeit other *Bordetella* bacteria such as *B. parapertussis* and *B. holmesii* [22] can cause less severe symptoms. Accurate and timely diagnosis of pertussis is extremely important, but the diagnosis of pertussis and accurate laboratory detection of *Bordetella* infections is still challenging. For instance, the commonly used culture of *B. pertussis* is fastidious, with limited sensitivity [23], while previously developed PCR-based methods of *B. pertussis* have been confirmed with the shortage of low sensitivity or cross-reaction with other *Bordetella* bacteria strains [8, 9, 12, 13].

In this study, MCDA-LFB based on the PT promoter region of *B. pertussis,* with excellent specificity, was successfully established. The high specificity of *B. pertussis*-MCDA-LFB is likely due to applying PT promoter region as detection target [12, 13]. Furthermore, the primer set based on different sequence of the promoter region also contributed the high degree of specificity of *B. pertussis*-MCDA, which has been previously demonstrated [24]. Particularly, the MCDA primer set, which recognized 10 regions of PT promoter, also ensured the assay’s specificity. Although several closely related species (such as *B. bronchiseptica*, *B. holmessii* and *B. hinzii*) did not be examined to validate B. pertussis-MCDA-LFB’s specificity, the data of specificity from NCBI BLAST has revealed that the MCDA primer set designed here was specific to *B. pertussis*. Furthermore, the specificity of the assay was further evaluated by using non-*B. pertussis* strains (Fig. 6) and clinical samples (Table 3). Therefore, the *B. pertussis*-MCDA-LFB established in the present study is of high degree of specificity.

Except the advantage of specificity, the *B. pertussi*s-MCDA-LFB established in the present study also displayed excellent sensitivity. The detection limit for *B. pertussis* pure cultures reached as low as 10 fg DNA per reaction (Fig. 4). Compared with the official real-time PCR assay recommended by China CDC, the *B. pertussi*s-MCDA-LFB displayed better sensitivity because the limit of detection of real-time PCR kit was 70 copies per reaction (equivalent to 292 fg per reaction) provided by its manual. Furthermore, the application of detection for *B. pertussis* in clinical samples also verified the outstanding sensitivity of the *B. pertussis*-MCDA-LFB, which yielded higher positive rates than real-time PCR assay (Table 3). The lower detection rate of real-time PCR may be due to the factors that the copy numbers of the *B. pertussi*s templates were lower than LoD (limit of detection), or the presence of some inhibitors specific to real-time PCR decreased the reaction sensitivity.

Fast diagnosis of the pathogenic agents contributes to the accurate clinical diagnosis and rational therapy of patients. Traditional culture method needs more than 1 week to determine *B. pertussis* in the clinical sample, while serological diagnosis needs about 1 month [7, 25]. MCDA-LFB assay developed in this study can be finished within 70 min, which includes 25 min for sample preparation, 40 min for amplification reaction and 2 min of results judgment. Lateral flow biosensor used for result interpretation in this study is time-saving, simpler compared with other methods such as traditional culture, PCR based methods or LAMP. Besides, result interpretation with LFB is based on the test line and control line, thus it is more objective and less error-prone compared with traditional culture, PCR based methods or LAMP.

Detection cost may be an economic burden for many laboratories, particularly in developing countries, which may influence the timely diagnosis for patients. The *B. pertussis*-MCDA-LFB merely needs a simple incubation at 64 °C, because MCDA-LFB is independent of thermal denature and temperature changes during the reaction. Therefore, it has extensive application prospects in resource-poor laboratories, which lack the advanced and precise equipment. Besides, each MCDA-LFB test only costs $5.5 USD, including bout $3.5 USD for reaction and $2 USD for LFB. Additionally, Certified technical personnel are not the prerequisite for the operation of MCDA-LFB due to its easy and simple operation, so that the labor costs are decreased. Therefore, *B. pertussis-*MCDA-LFB assay exhibited distinct strength in terms of costs for equipment, labor, experimental reagents and material. Notably, our *B. pertussis*-MCDA-LFB assay used similar sample collection and template extraction techniques as the B. pertussis-PCR and RT-PCR assays, it is subject to the potential shortcomings with regard to the availability of extraction kits, extraction instruments and reagents.

## Conclusions

*B. pertussis*-MCDA-LFB was successfully developed for the effective detection of *B. pertussis*. It exhibited strength of specificity for the detection of *B. pertussis*, with detection limit of 10 fg of DNA from pure culture of *B. pertussis*. It can be finished within 60 min, with convenient and simple operation and it does not require costly equipment. Thus, the *B. pertussis*-MCDA-LFB assay offers an effective strategy for rapid detection of *B. pertussis,* which is of potential application value in the field and resource-poor laboratories.

## Methods

### Reagents and instruments

The reagents of Malachite Green (MG) and Isothermal Amplification Kits were ordered from BeiJing-HaiTaiZhengYuan Technology Co., Ltd. (Beijing, China). DNA Extraction Kits were purchased from SBS Genetech Co., Ltd. (Beijing, China). Crimson red and streptavidin coated polymer nanoparticles were obtained from Bangs Laboratories, INC. (Indiana, USA). Biotinylated bovine serum albumin (Biotin-BSA) and rabbit anti-fluorescein antibody (Anti-FITC Ab) were obtained from the Abcam Co., Ltd. (Shanghai, China). Nitrocellulose (NC) membranes, membrane backing materials, sample pads, conjugate pads and absorbent pads were produced by the Jie Yi Biotechnology Co., Ltd. (Shanghai, China).

### Nanoparticle-based biosensor

Lateral flow biosensor (LFB, 4 mm × 60 mm) used in this study was prepared according to previously reported operation with some modifications [26]. Briefly, a backing card was laminated with a sample pad, absorbent pad conjugate pad and NC membrane. The test line (TL) and control line (CL) were prepared by spraying NC membrane with anti-FITC Ab (0.25 mg/ml) and biotin-BSA (2.5 mg/ml), with separation of 5 mm between the TL and CL. The conjugate pad of the strip was then coated with streptavidin which was coated with polymer nanoparticles resolved in 0.01 M PBS (PH 7.4). The prepared cards were sliced into 4-mm-wide strips (Deli No. 8012) and packed with plastic bag accompanied with desiccant gel and stored at RT (room temperature).

### Genomic DNA template preparation

*B. pertussis* strains were cultured on charcoal agar plates (OXOID, UK) supplemented with15% of defibrinated sheep blood at 37 °C for 3 to 5 days [27]. DNA Extraction Kit (SBS Genetech, Beijing, China) were used to prepare the DNA template based on the operation instruction. DNA was quantified with the instrument Nano drop ND-1000 (Calibre, Beijing, China). 100 ng/μL of target DNA was used for a 10-fold dilution (1 ng/μL, 100 pg/μL, 10 pg/μL, 1 pg/μL, 100 fg/μL, 10 fg/μL and 1 fg/μL), which were equivalent to 2.4 × 10^5^ copies/μL, 2.4 × 10^4^ copies/μL, 2.4 × 10^3^ copies/μL, 2.4 × 10^2^ copies/μL, 2.4 × 10^1^ copies/μL, 2.4 × 10^0^ copies/μL and 2.4 × 10^− 1^ copies/μL. In particular, the number of genomic copies for each dilution was confirmed based on the fact that 4.17 fg of genomic template was equivalent to a single genome of *B. pertussis* (assuming a molecular size of 4.1 Mbp for *B. pertussis*) [28, 29]. The serial dilution of genomic DNA of *B. pertussis* strain ATCC-9340 was used to test the sensitivity of *B. pertussis*-MCDA-LFB. Non-*B. pertussis* strains were mixed with 10% (w/v) glycerol broth and kept at − 70 °C. The genomic DNA of non-*B. pertussis* strains (Table 2) were extracted with the QIAamp DNA Mini Kit (Qiagen, Germantown, MD, USA).

### *B. pertussis*-MCDA primer set design and synthesis

The PT promoter sequence targeting *B. pertussis* was applied for MCDA primer design. A set of 10 primers (F1, F2, CP1, CP2, C1, C1*, C2, D1, D2, R1* and R2) was designed by using PREMIER 5.0 to establish MCDA assay. Integrated DNA Technologies design tools were applied to analyze the hairpin structures and hybrids of primer sequences. Specificity of the MCDA primers for *B. pertussis* detection was confirmed by blast analysis in NCBI data base. The biotin and fluorescein isothiocyanate (FITC) was used to label the 5′ ends of C1 and R1 primer, individually. The primer information is shown in Fig. 1 and Table 1. All the primers of HPLC purification grade were produced by Tianyi-Huiyuan Biotech Co., Ltd. (Beijing, China).

### The *B. pertussis*-MCDA reaction

MCDA reactions were performed in 25 μl reaction system as previously described [30]. The reaction system for each sample contained 12.5 μl of 2 × reaction mix (isothermal amplification® kit), 0.4 μM of primer F1 and F2, 0.8 μM of primer C1^*^, C2, R1*, R2, D1 and D2, 1.6 μM of cross primer CP1 and CP2, 1 μl of *Bst* 2.0 DNA polymerase (8 U) and 1 μl of DNA template. MG and LFB detection were simultaneously applied to monitor the MCDA amplification products. By using the MG method, color changes from colorless to light green as a result of the reaction products should be detectable while no color changes observed in the negative and blank control. When using the LFB for product detection, two visible lines should be observed in positive reactions at the location of CL and TL, respectively, but only one line at the location of CL appear in the blank controls and negative. By confirming the target-specific formation of MCDA products by electrophoresis gels images, a DNA ladder should be observed in positive reaction, but no DNA ladder observed in the negative and blank control.

### Optimizing the amplification temperature of *B. pertussis*-MCDA-LFB

Real-time turbidity determination was used to monitor the MCDA reactions for detecting the DNA *B. pertussis*. The optimum reaction temperature was determined using DNA of *B. pertussis* from 60 °C to 67 °C for 60 min, with interval of 1 °C. Turbidimeter (LA-320C) was used to monitor the turbidity of MCDA amplification. Genomic DNA (1 μl) of *S. aureus* and *N. meningitidis* strains were used as negative controls, and double-distilled water (DW) were chosen as blank controls.

### *B. pertussis*-MCDA-LFB sensitivity determination

The serial dilutions of of B. *pertussis* genomic templates described above were used to determine the limit detection of B. *pertussis*-MCDA assay. LFB and colorimetric indicator (MG) were used to detect the MCDA amplification results, respectively.

### Optimizing the amplification time of *B. pertussis*-MCDA-LFB

The serially diluted DNA was used to optimize the MCDA reaction time. MCDA reaction mixture was incubated for 10, 20, 30 or 40 min at the optimal temperature. The MCDA amplification products were detected with a LFB, Test of each amplification time were repeated at least two times.

### *B. pertussis*-MCDA-LFB specificity determination

The genomic DNA templates of 17 *B. pertussis* strains and 27 non-B*. pertussis* strains were applied to validate the specificity of *B. pertussis*-MCDA. LFB was used to detect the amplification results (Table 2). The examinations for MCDA specificity validation were repeated at least two times.

### Application of the *B. pertussis*-MCDA-LFB in clinical samples

A total of 67 nasal swab samples, collected from patients distributed in the prefecture Guiyang, Anshun, Zunyi, Tongren, Qiannan, Qiandongnan Qianxinan and Bijie of Guizhou Province, were used for the clinical validation of *B. pertussis*-MCDA-LFB. Particularly, the clinical samples used in this study, which were part of the routine CDC (Center for Disease Control and Prevention) laboratory procedure in China, were not specifically isolated for this research. The National Health and Family Planning Commission of China determined that the collection of data from human cases of infectious disease was part of continuing public health surveillance of a notifiable infectious disease and was exempt from institutional review board assessment [31]. All data were supplied and analyzed in an anonymous format, without access to personal identifying information.

The total genomic DNA templates from the clinical samples stored in − 70 °C for further use after regular detection by traditional culture and a real-time PCR assay (Recommended by China CDC) were used for the evaluation of *B. pertussis*-MCDA-LFB in clinic detection. The specificity and sensitivity of *B. pertussis*-MCDA-LFB, traditional culture and the conventional real-time PCR for the detection of the genomic DNA of *B. pertussis* from the clinical samples were compared. In particular, the *B. pertussis*-RT-PCR assay recommended by China CDC (MABSKY Biotech Co., Ltd., Beijing, China) was conducted according its manual (^#^SKY-PCR-FZ-300-1). Briefly, each 25 μL reaction mix contained 12.5 μL of 2× Reaction Mix, 2 μL of primer mix, 5.5 μL of deionized water and 5 μL of templates. Amplification was carried out as the following conditions: 95 °C for 3 mins, followed by 40 cycles of 95 °C for 5 s, 55 °C for 40 s.

## Data Availability

The datasets supporting our findings are included in the article.

## Abbreviations

MCDA: Multiple cross displacement amplification
PT: Pertussis toxin
DNA: Deoxyribo Nucleic Acid
IS: Insertion sequences
LAMP: Loop-mediated isothermal amplification
MG: The reagents of Malachite Green
NC: Nitrocellulose
TL: Test line
CL: Control line
NCBI: National Coalition Building Institute
FITC: Fluorescein isothiocyanate
HPLC: High Performance Liquid Chromatography
ATCC: The Global Bioresource Center
GZCDC: Guizhou Provincial Center for Disease Control and Prevention
DW: Distilled water
WHO: World Health Organization
USD: United States dollar
